# Rhegmatogenous retinal detachment surgery: A review

**DOI:** 10.1111/ceo.14205

**Published:** 2023-01-25

**Authors:** Alexis Warren, Daniel W. Wang, Jennifer I. Lim

**Affiliations:** The University of Illinois at Chicago, Department of Ophthalmology, Chicago, Illinois, USA

**Keywords:** retinal detachment, scleral buckle, subretinal fluid, vitrectomy

## Abstract

Rhegmatogenous retinal detachment (RRD) is a serious surgical condition with significant ocular morbidity if not managed properly. Once untreatable, approaches to the repair of RRD have greatly evolved over the years, leading to outstanding primary surgical success rates. The management of RRD is often a topic of great debate. Scleral buckling, vitrectomy and pneumatic retinopexy have been used successfully for the treatment of RRD. Several factors may affect surgical success and dictate a surgeon’s preference for the technique employed. In this review, we provide an overview and supporting literature on the options for RRD repair and their respective preoperative and postoperative considerations in order to guide surgical management.

## 1 | INTRODUCTION

Achievement of excellent anatomic and visual outcomes following repair of retinal detachments requires careful preoperative evaluation and planning, skilled intraoperative closure of the causative retinal breaks, postoperative patient compliance and intrinsic ocular factors. The risk of postoperative proliferative vitreoretinopathy encompasses preoperative ocular factors and intrinsic cytokine profiles. Scleral buckling, pars plana vitrectomy (PPV) and pneumatic retinopexy are all capable of delivering excellent outcomes when performed in appropriate scenarios. This article will focus upon the preoperative, operative and postoperative factors and appropriate scenarios for these procedures.

PPV has become increasingly utilised and has largely become the standard of care for treatment of rhegmatogenous retinal detachment (RRD). Vitrectomy now accounts for more than 70% of procedures performed for RRD repair in the United States, while the utilisation of scleral buckle surgery continues to decline. In 1980, PPV accounted for merely 1% of all RRD repair; by 2014 this percentage had increased to 83%.^1,2^ Regardless of which technique is used for retinal detachment repair, the success of the surgery hinges on localising and treating all retinal breaks. The advent of wide-angle viewing systems contributed to the popularity of vitrectomy repair as the surgeon’s ability to visualise peripheral pathology was markedly improved and undoubtedly contributed to the successful results. Advances in the technology and technique of PPV have permitted increased patient comfort and hastened postoperative recovery.^3^ In contrast, there have been relatively few advances in scleral buckling procedures. Nonetheless, scleral buckling is a valuable, effective and less invasive procedure for retinal detachment repair. Pneumatic retinopexy is yet another effective and even less invasive surgical option for repair of retinal detachment. Of course, pneumatic retinopexy is a viable option for only specific types of retinal detachments, primarily based on the number, location and size of the retinal tears and absence of proliferative vitreoretinopathy. The selection of which procedure to use involves consideration of patient characteristics, ocular comorbidities (e.g., high myopia, scleral ectasia, lenticular status) and of course characteristics of the retinal detachment itself.

## 2 | PREOPERATIVE CONSIDERATIONS

The preoperative examination and counselling process is critical. Assessment of a patient’s ability to position after surgery must be considered. Various rheumatological, degenerative neck/back and cardio/pulmonary conditions may prohibit persistent facedown or specific positioning (e.g., head tilted to a specific side). Duration of head positioning varies with the procedure and with the underlying diagnosis. Early postoperative positioning (initial 24 h) is often important for subretinal fluid management and to avoid complications such as folds in the macular region. Often patients are placed into the prone, head-down position immediately following a vitrectomy procedure and before going to the recovery area. Encouragement of the prone position is emphasised and maintained for one full hour prior to discharge from the hospital. It has been suggested that face-down positioning is associated with a reduction in the rate and amplitude of postoperative retinal displacement after macula-involving retinal detachment repair.^4^ Adjustable postoperative positioning with alternative upright or lateral recumbent has demonstrated similar efficacy to strict facedown positioning in select patients.^5^ In patients undergoing pneumatic retinopexy, the angle of positioning is specific to the location of the tear. In these patients, a forward tilt to avoid gas touching the lens combined with the specific side (right or left) to tilt the head is necessary. In these case, one can draw an arrow on the patient’s forehead that will point upwards if the patient’s head is tilted in the desired direction. The patient and the family members are instructed to position the patient’s head with the arrow pointed to the ceiling to get the best outcome. Adherence to postural positioning after gas tamponade is higher with female patients than male patients with diurnal variation.^6^ Although a few case series have demonstrated good anatomical success with limited <24 h postoperative positioning, we prefer at least 1 to 2 weeks of strict positioning.^7,8^ In general, recommend face down positioning if the gas bubble is >30% to limit cataract formation.

Consideration must be given to any occupational requirements and the potential (e.g., face up position for electricians) for high altitude or air travel as it may dictate the choice of intraocular tamponade. As part of the informed consent process, the possible need for additional surgery including the risks of cataract formation in phakic patients should be discussed. This rate of cataract progression and formation exists and is similar to 20-gauge systems: 96% after 20-gauge and 72% after smaller gauge.^9^ A pre-existing cataract as well as increased age raises the odds that cataract surgery will be required sooner. By performing a combined surgery (phaco-vitrectomy), the patient may be spared from an inevitable second procedure with its associated risks, costs and postoperative recovery process. Furthermore, removing a pre-existing cataract prior to vitrectomy may enhance intraoperative visualisation, facilitate a thorough shave of the peripheral, anterior vitreous and aid in achieving a more complete gas tamponade.

Preoperative examination of the sclera is warranted to determine whether areas of scleral thinning or ectasia are present. The presence of severe thinning raises the risk that scleral perforation could occur during scleral buckling procedures. Of course, intraoperative examination of the sclera should also be done prior to cryotherapy to detect similar areas of scleral thinning.

In situations where the natural lens impairs a thorough retinal evaluation to determine the presence of retinal breaks or in which significant portions of the retina are not visible, PPV is selected over scleral buckling or pneumatic retinopexy procedures. Similarly, in eyes with significant amounts of vitreous haemorrhage that obscure the peripheral retina, PPV is the better option.

Most vitrectomies are performed under monitored care anaesthesia with a retrobulbar block unless the patient is unable to cooperate during surgery; 61% of surveyed retina specialists in the United States prefer a retrobulbar injection whereas 20% perform a conjunctival cutdown and sub-tenon’s block.^10^ In children, patients with disabilities that cannot lie still with local anaesthesia or in those with a significant history of claustrophobia, general anaesthesia is needed.

## 3 | OPERATIVE PROCEDURES

### 3.1 | VITRECTOMY

#### 3.1.1 | Vitreous removal

The 25-gauge vitrectomy set-up offers both adequate fluidic/tissue stability and instrument performance/rigidity and might be the preferred choice for almost all cases. Rates of reattachment and functional outcomes are comparable between 27-gauge and 25-gauge vitrectomy; however, there is a suggestion that surgical time is longer using 27-gauge systems.^11^ The position of the identified breaks and the location of the detachment does not influence where the trocars are placed. However, we do not hesitate to move the infusion port location to better access the retinal pathology as needed. In general, we do suture sclerotomy sites at the conclusion of the case. This alleviates the requirement of beveling/tunnelling during trocar insertion and reduces risks of postoperative would leaks. Rates of hypotony and measured intraocular pressure do not differ between 27-gauge and 25-gauge groups.^12,13^

Caution should be taken, especially in cases of bullous retinal detachments, to not insert the infusion cannula into the subretinal space or to incarcerate any retinal tissue into the sclerotomies. The infusion is customarily placed in the inferotemporal quadrant to permit an adequate wider range of mobility when operating. If combined surgery is to be performed, the infusion cannula may be placed first before commencing phacoemulsification. The connection of the infusion line may be delayed until the PPV commences so that it is not get in the way during the phacoemulsification.

A core vitrectomy should be performed with attention to carefully remove the vitreous attachment to all retinal breaks. It is also imperative to clear the vitreous around the infusion cannula and superior sclerotomies to avoid vitreous incarceration which may occur, especially if one is using non-valved gauged vitrectomy systems.^14^ A complete posterior vitreous detachment (PVD) should be created if not already present. PVD should be extended as far anteriorly has possible (without creating iatrogenic tears) to ensure adequate vitreous removal. Iatrogenic breaks have been reported to be as high as 15.8% in a consecutive case series using 25-gauge sutureless vitrectomy.^15^ Dynamic scleral depression and visualisation of schlieren aid in identifying causative breaks and their location(s).^16^

It is advantageous to use the shave mode/mobile retina setting a low flow/aspiration state as this gives excellent control of the procedure and limits potential retinal incarceration into the vitrector.^17^ Scleral indentation to allow for careful vitreous base shaving 360 degrees is indicated. If a flap tear is present, it should be excised. Furthermore, diathermy with subsequent cauterization of any observed bridging vessels over the causative retinal breaks may be necessary to prevent any potential bleeding at the cut edge. Any vitreous traction to the detached retina should be relieved with removal of adherent vitreous or by scleral buckling support.

#### 3.1.2 | Laser retinopexy

Laser is typically employed to treat the retinal break(s) and cyrotherapy is avoided, particularly if many spots would be needed because extensive cryotherapy may increase the risk of epiretinal membrane formation by inducing retinal pigment epithelial cell release.^18^ By marking the posterior edge of all retinal breaks with diathermy, easier visualisation is achieved when the posterior cavity is air-filled. Two to three rows of laser spots around the retinal break is required to seal its edge. The spots may be confluent, or very closely spaced. A flexible, curved endoilluminator provides wider cone angles of illumination despite smaller light fibres. Additionally, the surgeon may perform depression to visualize the tear and apply laser retinopexy without the aid of skilled assistance by use of a chandelier endoilluminator. If there is subretinal fluid present, an air-fluid exchange to drain this fluid should be performed to ensure reattachment and adequate laser uptake. An advantage of performing laser retinopexy under air is that a wider field of view (but with reduced magnification) is achieved. There is in general no benefit for “prophylactic” 360-degree laser of the peripheral retina in the absence of large circumferential giant tears or 360-degree retinectomy. In fact, 360-degree laser retinopexy has been suggested to have lower final anatomic success and worse final visual acuity when controlling for case complexity.^19^

#### 3.1.3 | Drainage

Passive subretinal fluid drainage using a soft-tipped backflush may theoretically lead to less retina displacement and/or formation of outer retinal folds. Active aspiration with rapid retinal reattachment could lead to more outer retinal folds. This concept of passive subretinal fluid removal has been theorised by Muni et al.^20^ The eye should be turned so the retinal break is the deepest, most dependent possible point of the eye. The backflush should be placed just anterior to the posterior edge of the retinal break. When bubbling is heard during the air-fluid exchange, only air is being removed, and the instrument may be advanced towards the break to continue draining residual fluid. Once the subretinal fluid is sufficiently drained, additional drainage should be performed overlying the optic disk.

#### 3.1.4 | Tamponade

Although the usage of air has been described in the literature in achieving favourable primary or overall anatomic success for superior pathology,^21^ we prefer longer-acting tamponade agents. Perfluorooctane (C3F8), gas is generally employed for most cases, particularly if retinal breaks are below the 4 and 8 o’clock meridian. This ensures adequate retinal tamponade of the breaks by a large enough gas bubble that contacts the inferior breaks while the laser retinopexy adhesions seal. This is also helpful for patients who may not position fully face down as directed. We also use sulphur hexafluoride (SF6), for smaller breaks and for detachments which are less likely to progress to proliferative vitreoretinopathy, particularly those with small breaks above the horizontal meridian. In general, gas tamponade has been found to be superior to air tamponade in detachment cases with involvement of lower quadrants.^22^ Air tamponade should only be reserved in selected cases where pathology is restricted to the superior quadrants. For redetachments and detachments secondary to proliferative vitreoretinopathy and cases treated with retinectomy, silicone oil placement should be strongly considered as it yields significantly higher anatomical and surgical success than shorter-acting gasses and for those patients who cannot assume face-down positioning or for those patients who must travel to high altitudes.^23^ The silicone oil study demonstrated that silicone oil was superior to SF6 but equal to C3F8 in achieving anatomic success of retinal detachment repair with proliferative vitreoretinopathy.^24,25^ All patients should be informed that in addition to air travel, the use of gas tamponade, restricts any activity where there are may be shifts in atmospheric pressure including scuba diving and mountain climbing. Immediately following surgery, all patients should wear a wristband indicating the presence of an intraocular gas tamponade. While a gas tamponde is still present, patients should not receive nitrous oxide gas, a common anaesthetic with anxiolytic properties widely utilised in clinical settings. In the presence of an intraocular gas tamponade, because nitrous oxide diffuses more readily into an air-fluid cavity, there may be a rapid elevation in intraocular pressure potentially leading to significant ocular morbidity.

### 3.2 | Additional considerations

#### 3.2.1 | Perfluorocarbon fluid

Perfluorocarbon liquid (PFO) has been a major milestone in vitrectomy surgery and is an invaluable tool in the repair of giant retinal tear-associated detachments.^26^ By stabilising the mobile, detached retina, PFO reduces the risk of iatrogenic breaks, especially towards the periphery. PFO may also be useful to assist with subretinal fluid drainage in cases when the retinal break is anterior in order to avoid the need for a drainage retinotomy. When instilling PFO, a double-bore soft-tip cannula may be employed. To minimise the risk of subretinal migration, the PFO should be injected slowly, with the tip of the cannula overlying the posterior pole or optic nerve. One should avoid its use in the presence of posterior breaks. All retinal traction should be removed prior to PFO injection, and the fill may continue until it reaches the posterior edge of the anterior break. This will permit subretinal fluid drainage with a cannula through that opening to flatten the retina. An air-fluid exchange is performed during which the subretinal fluid is sandwiched between the air bubble and the PFO. The brushed tipped cannula is used to aspirate the subretinal fluid through the retinal break. Meticulous, careful removal, however, should be taken to avoid retained PFO at the conclusion of the case. Lavage of the retinal surface using a balanced salt solution (BSS) may assist with the removal of residual droplets. The incidence of subretinal PFO is between 0.9% and 11.1% and most commonly observed in eyes with large peripheral retinotomies and eyes that did not undergo a thorough saline rinse.^27,28^

#### 3.2.2 | Retinectomy or retinotomy

Two major indications for retinectomy or retinotomy include retinal shortening or retina with inseparable vitreous and/or membranes. One must carefully consider the location and length of the performed procedure. A thorough anterior vitreous base dissection must be performed to relieve anterior trough/retinal contraction. A lensectomy should be considered to permit anterior dissection. All circumferential puckering and pre-retinal membranes should first be removed. Diathermy should be applied, just posterior to where vitreous/membrane and retina separation was not possible or in areas without retinal fibrosis in cases of severe retinal foreshortening despite complete membrane excision. If the retinotomy is less than 360 degrees, the retinectomy edges should reach anteriorly towards the ora serrata. The edges of the retinectomy should be adjusted to ensure the release of all potential traction. In general, we add an extra clock hour beyond that needed to fully reattach the retina. This helps prevent the need for additional surgery as the retina typically contracts a little bit more in the postoperative period and this could lead to recurrent elevation at the retinectomy edges. Subretinal PVR should only be excised if the retina remains elevated locally due to the subretinal band of PVR. At the conclusion of the manoeuvre, PFO and an air-fluid exchange should be performed. Care should be taken to avoid inadvertent retinal translocation in cases where a 360-degree retinectomy is performed.

Retinotomy may be employed as the initial step in performing a retinectomy, but stand-alone indications also exist.^29^ If the retina is shortened circumferentially, a radial cut can relieve the traction. A small retinotomy may also be considered in times where access to the subretinal space needs to be obtained to drain fluid or remove a subretinal PVR membrane that is tenting the retina. The anterior flap of the retinotomy is avascular and non-functional. Excision of the retina is recommended so that fibrin and cellular proliferation do not contribute to inflammation or neovascular proliferation from the anterior flap which could result in traction on the ciliary body.

#### 3.2.3 | Combined PPV with scleral buckle

In cases where the causative pathology is primarily inferior and intraocular gas tamponade is unlikely to provide effective tamponade, a combined vitrectomy and scleral buckling procedure may be considered. The PRO Study Report 9 demonstrated that combined PPV/SB had superior anatomic outcomes compared with primary in RRDs with inferior retinal breaks, particularly in phakic eyes.^30^ Supplementary scleral buckling may enhance primary surgical success in inferior detachments over conventional PPV with gas alone.^31^ In most cases of combined PPV and SB, the encircling band should be placed prior to vitrectomy to ameliorate the technical challenges of working on a non-firm eye with fluctuating intraocular pressure due to vitrectomy port placement and connection to the vitrectomy machine. In phakic and pseudophakic patients with moderately complex RRD, scleral buckle and PPV combined were superior to PPV alone in single-surgery anatomic success rates.^32,33^

## 4 | SCLERAL BUCKLE

The premise of scleral buckling is based upon the fundamental principle that reattachment of the retina requires not only sealing of the breaks, but relief of the associated traction, which can be achieved through increased apposition of the retinal pigment epithelium and choroid. The technique used for the relief of this traction was trialled by several pioneering surgeons including Strampelli, Custodis and Schepens who first used scleral shortening procedures, blood plasma and even gauze pads to achieve the support of the retinal tears.^34^ Scleral buckling has proven very successful in primary rhegmatogenous detachment repairs with success rates as high as 95%, even at 20 year follow-up.^35^ Unfortunately, since the invention of small gauge PPV with similar single repair reattachment rates, scleral buckling procedures have been less often utilised for primary retinal detachment repair. Reasons for this are likely multifactorial and include more difficult visualisation of the retina compared to wide-angled viewing, more time and skill required to isolate and support the retinal break compared with vitrectomy and more potential risk and complexity required to drain subretinal fluid than with an air-fluid exchange. These reasons combined with decreased exposure of recent trainees to the procedure have led to declining numbers of scleral buckling procedures. The Wilmer Eye Institute worked to quantify these changes in practice patterns and recently published their 10-year data for the management of uncomplicated retinal detachments. This showed a change from a predominance of SB procedures in 2008–2011 to that of PPV by 2015–2017.^36^ Despite this change, the number of eyes that underwent combined PPV and scleral buckling remained stable over time.^36^ This change in surgical decision-making proved to be driven primarily by surgeon choice and not by patient and detachment characteristics.^36^ Indeed, surgeons who were more early in their careers were more likely to choose PPV over scleral buckling when compared with their more senior colleagues.^36^ Still the debate remained whether these choices resulted in any significant difference in visual outcomes for patients.

Some of the most recent data from meta-analysis in 2021 by Dhoot et al noted scleral buckles to have greater visual outcomes when compared with PPV (20/48 vs. 20/43 Snellen), although this is unlikely to be clinically significant.^37^ This study also found that scleral buckles have a statistically significant lower incidence of cataract formation and iatrogenic breaks, as expected, and as such might be a reason to prioritise this type of repair in the correct patient.^37^ Despite the lack of standardised parameters to guide in which scenarios one repair technique might be superior to the other, there does appear to be subset of patients who may have higher success rates for reattachment using a scleral buckle. These factors include patients with significant inferior pathology, including multiple tears or extensive lattice degeneration. Data from as recent as the 2022 American Society of Retinal Specialists (ASRS) Preferences and Trends Survey (PATS) still points to scleral buckles as the primary surgery of choice (48.2% SB vs. 23.2% for PPV, and 27.4% for PPV/SB) for participating retinal specialists in the United Sates in the repair of inferior phakic primary retinal detachments.^10^ This notion is further supported by one of the largest primary scleral buckle and vitrectomy trials to date by Heimann et al. In this study, there was statistically significant (*p* = 0.0005) and greater best corrected visual acuity (BCVA) improvements in phakic patients who underwent a scleral buckle (−0.71 logMAR vs. −0.56 logMAR for PPV).^38^ However, additional subgroup analysis of pseudophakic eyes did not show any significant difference in the change in BCVA between the two procedures.^38^ Despite this, scleral buckles have previously been considered an inferior option for the repair of pseudophakic primary detachments. Several randomised control trials have also previously quoted statistically significant and better primary reattachment rates for PPV compared to SB.^38,39^ Yet, more recent analysis may prove this to be a false narrative. The most current large-scale meta-analyses of subgroups including lens status, macula-on/off status and even location of the break have shown no difference in primary reattachment rates between these two procedures.^37^ There does however appear to be better anatomic success in combined scleral buckle vitrectomy procedure for the more extensive and macula off cases (*p* = 0.03).^40^

Various scleral buckle elements are currently available, most are made of silicone rubber, which is inert and less prone to infection or extrusion compared to the historically used polyethylene or hydrogel buckles.^34,41^ Encircling bands are a great option, especially in cases with multiple tears requiring support, but radial elements which provide more focal indentation to single breaks and can also be used.^42^ Prior to securing the scleral buckle implant, one must treat the retinal break(s) using retinopexy, most often using transscleral cryotherapy. Indirect laser therapy may also be used however, this is much less often employed, despite having similar success rates.^43^ Visualisation for retinopexy in the operating room has traditionally required using the indirect ophthalmoscope. However, this may be complicated especially given the limited field of view and small pupils. Thus, the concept of wide-angle non-contact viewing has gained increasingly popularity. The use of a single 25-gauge cannula and chandelier endoilluminator allows for both magnification and focusing power and may be one way to overcome visualisation challenges without significant disruption to the vitreous cavity. As this has become more readily accessible to surgeons there has been increasing literature to support its non-inferiority to standard indirect viewing. To date, chandelier assisted scleral buckle repair has shown primary reattachment rates ranging from 78% to 95% which is at least equally favourable to our conventional repair methods.^44–46^ Unfortunately, more data are needed to see if the possible complications from disturbing the vitreous (i.e., prolapse and iatrogenic breaks) may make this technique less advantageous than it would appear.

## 5 | PNEUMATIC RETINOPEXY

The concept of pneumatic displacement with supplemental retinopexy for primary retinal detachment repair was trialled and attempted for several years prior to its routine use by the retinal surgeon. It was not until 1985 when George Hilton and others re-introduced this technique as a unique in office procedure with single operation reattachment rates even close to vitrectomy surgery.^47^ However, since this time its use in everyday practice has been variable depending on many factors including surgeons experience, patients’ age and what is geographically accepted as standard practice.^48^ Yet, more recent data have shown that repair by pneumatic retinopexy may have other important advantages such as better visual acuity outcomes and self-reported vision-related quality of life.^49^ Hillier et al published one of the largest prospective randomised trials comparing PPV to pneumatic retinopexy (PnR). In this study, visual acuity after PnR was better than PPV by 4.9 letters at 12 months, which was statistically significant. These differences in best-corrected visual acuity were present even as early as 3 and 6 months.^49^ Accordingly, single surgery reattachment rates for pneumatic retinopexy have been similar to other repair techniques like scleral buckles.^50^ However, more recent data may prove an even larger gap with single operation success rates at 81%, for pneumatic retinopexy compared with 93% for repair by PPV.^49^ Although many surgeons would still consider this high reattachment rate a reasonable option for the correct patient, pneumatic retinopexy still remains less often utilised, especially in the age of smaller gauge surgery and easy access to ambulatory surgery centres.^51^ Interestingly, rates of pneumatic retinopexy are associated with weekends and some retina surgeons have described its use as a temporising procedure when access to the operating room is limited.

## 6 | POSTOPERATIVE

Head positioning is a key component in achieving tamponade of the retinal breaks as described earlier in this article. Successful outcomes also require careful postoperative monitoring to detect intraocular pressure elevation and to treat this elevation with pressure-lowering agents. Of course, patient counselling to avoid air travel or mountain travel while intraocular gas is present is also necessary. Patients with intraocular silicone oil may also require avoidance of face up positioning at bedtime depending upon the anterior segment status. Counselling to avoid strenuous activity and rapid head movements is also reasonable as the retinal laser or cryotherapy treatment heal to form permanent adhesions. Careful patient instruction as to the use of and need for compliance with postoperative medications is necessary.

More recently, methotrexate use as a postoperative adjuvant to limit the development of proliferative vitreoretinopathy has been used and studied in a clinical trial.^52–54^ The GUARD Study has shown positive outcomes with fewer patients developing recurrent proliferative vitreoretinopathy than untreated patients and historic controls. This may, in the near future, serve as treatment for patients with proliferative vitreoretinopathy retinal detachments if approved by the Food and Drug Administration (FDA). Whether prophylactic usage of this, or agents in the future, to prevent the development of proliferative vitreoretinopathy in high-risk patients, becomes the standard of care remains to be determined.

## 7 | SUMMARY

Given the relatively high reattachment rates across all repair techniques, greater emphasis has been placed on analysis of retinal displacement after surgical repair and its relation to patient satisfaction and functional outcomes. Recent meta-analysis shows retinal displacement rates after detachment repair as high as 35 ± 20%.^55^ However, the rate of displacement as assessed with fundus autofluorescence imaging differed depending on the surgical repair technique with primary scleral buckle having the lowest rate of displacement, followed by pneumatic retinopexy and PPV.^55^ More specifically, pneumatic retinopexy patients have shown less objective vertical and horizontal metamorphopsia compared to PPV patients. Accordingly, these pneumatic retinopexy patients show superior scores on the functional visual score questionnaires.^49^ These data points may highlight the need for a modification in our concept of successful detachment repair to one more focused upon patient-centred outcomes and less on anatomic measures. This shift is now possible because all surgical procedures are capable of delivering excellent anatomic outcomes when applied to patients with appropriate preoperative indications.
